# Predictive Screening for Inflammatory Disorders of Pregnancy Using Targeted Maternal Cell-Free RNA Assays: Proof-of-Principle Data from Large Animal and Human Cohorts

**DOI:** 10.1007/s43032-025-01876-w

**Published:** 2025-06-04

**Authors:** Sean W. D. Carter, Qin Wei, Winston Koh, Xiawen Liu, Kay Yi Michelle Seah, Si En Poh, Haruo Usuda, Erin L. Fee, Yusaku Kumagai, Tsukasa Takahashi, Lara Monteiro, Reyna Peñailillo, Hannah R. S. Watson, Masatoshi Saito, Owen B. Spiller, Mahesh A. Choolani, Sebastián E. Illanes, Matthew W. Kemp

**Affiliations:** 1 Department of Obstetrics and Gynaecology, Yong Loo Lin School of Medicine, National University of Singapore, 1E Kent Ridge Road NUHS Tower Block, Level 12, Singapore, 119228 Singapore; 2 Institute of Bioengineering and Bioimaging, Agency for Science, Technology and Research, 31 Biopolis Way, The Nanos, #07-01, Singapore, 138669 Singapore; 3 School of Pharmaceutical Sciences, Guangzhou Medical University, Guangzhou, Guangdong China; 4 Women and Infants Research Foundation, Perth, WA Australia; 5 Centre for Perinatal and Neonatal Medicine, Tohoku University Hospital, Sendai, Japan; 6 Division of Infection and Immunity, School of Medicine, Cardiff University, Cardiff, UK; 7 Reproductive Biology Program, Center for Biomedical Research and Innovation, Universidad de los Andes, Santiago, Chile; 8 IMPACT, Center of Interventional Medicine for Precision and Advanced Cellular Therapy, Santiago, Chile

**Keywords:** Cell-free RNA, Chorioamnionitis pre-eclampsia, Inflammation, Obstetric complications, Preterm birth, Antenatal tests, Minimally invasive

## Abstract

The management and prevention of key inflammatory-associated pregnancy complications such as chorioamnionitis and pre-eclampsia is hampered by a lack of early gestation risk screening tools. In a proof-of-principle study we used targeted cell-free RNA analyses of maternal plasma samples from large animal (sheep) and human pregnancy cohorts to develop a minimally invasive screening test for inflammatory markers. This study utilised a preterm sheep model of sterile and bacterial chorioamnionitis. Date-mated ewes received either intraamniotic Saline Control (*n* = 10) or *E.Coli* LPS (Sterile chorioamnionitis) with 2 days (*n* = 9) or 8 days exposure(*n* = 6). Preterm lambs were delivered at 124 ± 1d gestation. Findings were validated in a bacterial model of chorioamnionitis where ewes were exposed to 7 days of intraamniotic *M.Hominis* with delivery at 98 d gestion(*n* = 8) or 128d gestation(*n* = 8). Maternal blood was collected prior to intervention and at delivery in each group. Random Forest algorithm was used to analyse 8 cell-free RNA(cfRNA) targets related to inflammation in maternal plasma at baseline and delivery, identifying genes that separated animals with or without intrauterine inflammation. Plasma cfRNA data was compared to mRNA expression in placental tissue. Haematological and placental mRNA comparisons were analysed with ANOVA/Tukey HSD/Dunnett T3 tests. Maternal plasma cfRNA findings of intrauterine inflammation were then validated in human plasma samples from a cohort of patients with late onset pre-eclampsia (*n* = 10) or uncomplicated pregnancies (*n* = 10). We present data showing that targeted maternal cfRNA assays can accurately identify chorioamnionitis of sterile (AUC 1.0) and infectious (AUC 0.84) origin in a sheep model of pregnancy. Findings were then validated in human maternal plasma samples from patients with late-onset pre-eclampsia in the 1st (AUC = 0.85), 2nd (AUC = 0.90) and 3rd (AUC = 0.82) trimesters. In both sheep and human model systems, cfRNA tests offered high levels of sensitivity and specificity in the absence of overt clinical symptoms. We suggest that further development of this technology may serve as a scalable, rapidly deployed and cost-effective means for predicting major inflammatory conditions in pregnancy.

## Introduction

Pathological inflammation of the maternal and fetal tissues plays a key aetiological role in several serious obstetric complications, including chorioamnionitis (microbial or sterile inflammation of the gestational tissues), and pre-eclampsia (PE), a metabolic syndrome for which the key diagnostic features include hypertension and proteinuria occurring after the 20 th week of pregnancy. The global incidence of chorioamnionitis is estimated at around 4% with the highest rates found at extreme preterm gestations under 30 weeks’ gestation[1–4]; PE impacts between 3 and 5% of all pregnancies[5]. Together chorioamnionitis and PE account for a sizable percentage of the estimated 15 million preterm births (delivery prior to 37 weeks’ gestation) that occur each year, and over 1 million newborn deaths annually[6]. Importantly, both chorioamnionitis and PE are associated with sub-optimal fetal growth and/or adverse newborn outcome in survivors of preterm birth, including increased risk of respiratory diseases and neurological impairment [3, 6].

The pathogenesis of chorioamnionitis may be divided into infectious (detectable microbial invasion of amniotic cavity) or sterile (no detectable pathogen)[7]. The common sequelae of both being dysregulated intrauterine inflammation and a subsequent maternal–fetal inflammatory response. This dysregulated inflammatory response in pregnancy has been shown to induce cervical ripening, promote uterine contractions and result in increased likelihood of preterm birth^21^. Multiple clinical and animal studies have helped to better characterise this pathological inflammatory response in chorioamnionitis, demonstrating the presence of elevated levels of interleukins (IL)−1β, IL-6, IL8, IL-10 in amniotic fluid[1, 8–10]. However, corresponding elevated maternal plasma markers of inflammation (IL-1β, IL-2, IL-6, IFN-γ and TNF-α) have not been shown to be accurate for the diagnosis of chorioamnionitis[11]. On the other hand, inflammation also plays a key role in the pathophysiology of PE[12, 13]. This pathology begins early in pregnancy with abnormal trophoblast invasion of the spiral arteries, leading to uteroplacental ischemia and hypoxia[14]. Uteroplacental hypoxia is then proposed to result in the release of elevated levels of proinflammatory cytokines, endothelial damage and a systemic maternal inflammatory response[15]. Examples of abnormal cytokine levels associated with PE include elevated levels of ILs 1,2, 6, 8, 12, 18, tumour necrosis factor (TNF) α, increased circulating CD^14+^ cells and reduced levels of IL-10[14, 16]. Furthermore, cytokines such as TNFα have been shown to promote the secretion of soluble fms-like tyrosine kinase-1 (sFLT1), an antiangiogenic protein that sequesters placental growth factor (PlGF)[14]. An elevated sFLT1:PlGF ratio forms the basis of some current PE screening tests[17].

At present, the management of both PE and chorioamnionitis is hampered by a lack of robust screening methodologies with good positive and negative predictive values. The antenatal diagnosis of chorioamnionitis is most commonly based on clinical criteria alone (maternal fever, maternal or fetal tachycardia, uterine tenderness, foul smelling vaginal discharge as well as maternal leucocytosis) which have very poor sensitivity (56%) and specificity (55%) for the diagnosis of chorioamnionitis[18]. The poor predictive accuracy of these clinical criteria can lead to unnecessary and potentially harmful antenatal interventions, including administration of broad-spectrum IV antibiotics, antenatal corticosteroids and rapid iatrogenic delivery (often at preterm gestations) in women who end up not having histologically proven chorioamnionitis (i.e. false positive result). Conversely, in patients with a true positive diagnosis of chorioamnionitis, the clinical diagnosis is often only made once a very robust systemic maternal and fetal inflammatory response is present (i.e. stage IV chorioamnionitis). This late diagnosis then leads to late treatment initiation and delayed delivery, exposing both mother and fetus to increased risk of inflammation-driven morbidity and mortality. For the fetus, delayed diagnosis may result in the development of a systemic inflammatory response that has disastrous consequences including cerebral palsy, cerebral white matter damage, necrotising enterocolitis and death[19, 20]. Therefore, the early antenatal diagnosis of chorioamnionitis (prior to a systemic fetal and maternal inflammatory response) is critical to improving both maternal and fetal outcomes. At present the gold standard to confirm suspected chorioamnionitis is amniocentesis with culture and Gram stain (sensitivity 65%, specificity 99%, AUC 0.82)[21], or measurement of inflammatory markers including IL-6 (AUC of 0.84)[21]. However, amniocentesis is an invasive procedure[3] that requires technical skill and equipment that are not always readily available, and is associated with a small risk fetal loss[22, 23]. As such, no minimally invasive, highly accurate and rapid predictive test of subclinical chorioamnionitis exists at present.

In PE, the current approach for prediction and prevention is largely based on obstetrical history along with ultrasound and biomarkers (i.e. sFLT1:PlGF ratios). However, management based on historical risk factors are not applicable in pregnancies of nulligravida women, which constitute approximately 40% of deliveries. Accordingly, many groups have developed prediction models using biomarkers combined with maternal characteristics and uterine artery doppler velocimetry[24, 25]. Despite the clinical use of this algorithm, its poor predictive value leaves sizable room for improvement and development of new strategies for PE prediction.

Given that dysregulated inflammation plays an important role in the aetiology and pathophysiology of chorioamnionitis and PE, developing a method by which inflammatory markers in the maternal circulation may be measured as an early screening test may aid in the management of these important obstetric complications. To develop a preclinical test of inflammation in both chorioamnionitis and PE we set out to develop a minimally invasive predictive test of inflammation utilising targeted analysis of cell-free RNA (cfRNA) in maternal plasma. The purpose of this study was to use targeted cfRNA analyses of the maternal plasma transcriptome to perform a proof-of-principle assessment of screening for chorioamnionitis and PE in large animal and sequential human pregnancy cohort samples, respectively.

Two approaches were taken to assess the potential for maternal cfRNA analyses to screen for chorioamnionitis, both using the sheep model of pregnancy. Given the variability in human chorioamnionitis (i.e. sterile vs. infectious and various infectious subtypes) and the challenges in its diagnosis with regards to infection to diagnosis interval, we viewed that human samples would be extremely difficult to standardise, and thus have too much biological noise to support a proof-of-principle study. As sterile inflammation (no detectable intrauterine pathogen) accounts for a sizeable number of chorioamnionitis cases (bacterial pathogens are only detectable ~ 60% of the time in patients diagnosed with clinical chorioamnionitis[18]) we utilised a sheep model of pregnancy to expose the gestational tissues to intraamniotic endotoxin (lipopolysaccharides, LPS) from *Escherichia coli*, a well-validated model of sterile intrauterine inflammation. We additionally used the same sheep model of pregnancy to explore the ability of maternal cfRNA measurements to identify inflammation associated with intrauterine infection by *Mycoplasma hominis* (one of the most common chorioamnionitis-associated pathogens, accessing the gestational tissues via ascending vaginal infection)[3].

In the case of PE, we utilised a prospectively collected human pregnancy sample cohort for the exploration of cfRNA screening for PE risk. Aside from the obvious benefits of using a human sample cohort, this approach was selected as animal models were either unavailable or unsuited to this study. We are unaware of a sheep model of PE (likely due to differences in placentation), non-human primate models are based around an acute surgical model and murine knock-out models are impacted by their comparably short gestation. Thus, using human samples collected during the first, second and third trimesters of pregnancy allowed us to explore the utility of this approach in a human setting, whilst also confirming that the inflammatory screening panel also used for our sheep-based chorioamnionitis analyses worked in human samples (the ultimate objective of a screening test for chorioamnionitis and PE) from a technical perspective.

## Methods

### Experimental Model and Study Participant Details

#### Animal Model and LPS Treatments

In order to assess whether a discrete set of cfRNA targets related to inflammation could be detected in maternal plasma, and therefore be used to predict an intrauterine inflammatory insult, we undertook initial discovery studies utilising maternal samples from a sheep model of pregnancy. The animal ethics committee of the University of Western Australia reviewed and approved these studies prior to work commencing (Approval number 2022/ET000920) and adhered to the NIH Guide for the Care and Use of Laboratory Animals. Date-mated merino ewes with singleton fetuses received an intramuscular injection of 150 mg medroxyprogesterone acetate (Depo-Provera, Pfizer, New York, NY) on 117 ± 1 d of gestational age (dGA) to decrease the risk of intervention-induced premature labour.

Animals were then allocated to one of three interventional groups to undergo ultrasound guided intraamniotic injection (IAI) of either:

i) 2 ml sterile saline (Negative Control Group, n = 10) with delivery 8 d after IAI;
ii) 3.33 mg 055:B5 *E.coli* LPS (Cat# L2880, Sigma Aldrich Australia) (2 d Sterile Chorioamnionitis Group; n = 9) with delivery 2 d after IAI
iii) 3.33 mg 055:B5 *E.coli* LPS (Sigma Aldrich) (8 d Sterile Chorioamnionitis Group; n = 6) with delivery 8 d after IAI.

All animals were surgically delivered via hysterotomy at 124 ± 1 d gestational age (early preterm gestation, equivalent to 32 weeks’ human gestation). Lambs were weighed, subjected to necropsy, and extensively sampled, with tissues snap frozen in liquid nitrogen. Maternal plasma was collected immediately prior to lamb delivery into K3EDTA vacutainers, centrifuged at 1,500 × *g* for 10 min and frozen. The experiment was structured so that at least two animals from each of the two groups was delivered on each study day to assist in controlling for confounding. All animals were raised in a specific pathogen-free environment with stable temperature and humidity. In addition, animals were provided with free access to food and water under a 12 h light/dark cycle.

#### Sheep Intrauterine Infectious (*Mycoplasma hominis*) Inflammatory Exposure at both Extreme and Early Preterm Gestations

This study protocol was similar to the above LPS protocol and was likewise reviewed and approved by the animal ethics committee of the University of Western Australia prior to studies commencing (Approval number RA/3/100/1452).

Date-mated merino ewes with singleton fetuses at two different gestations of 91 d GA (extremely early preterm gestation equivalent to ~ 25 weeks human gestation) or 121 d GA (early preterm gestation equivalent to ~ 32 weeks human gestation) received ultrasound guided intraamniotic injections of either:

i) Sterile *Mycoplasma hominis* culture media (Media Negative Control Group; n = 4 per gestational age group); or
ii) *Mycoplasma hominis* bacteria at 2 × 10^7^ colour change units (CCU) (Infectious Chorioamnionitis Group; n = 8 per gestational age group). Surgical delivery of the fetus by hysterotomy occurred 7 days post exposure.

The *Mycoplasma hominis* isolate used was strain AH58, molecularly characterised in Chalker et al*.*[26]*.* The isolate used originated from a vaginal swab at 28 weeks gestation from a pregnant Australian woman who subsequently gave preterm birth. The study was approved by the Human Research Ethics Committee of the Western Australian Department of Health, Women and Newborn Health Service *(2056/EW)*[27]*.*

Animals exposed to either sterile media or *Mycoplasma hominis* at 91 d GA were delivered at 98 d GA. Animals exposed to either sterile media or *Mycoplasma hominis* at 121 d GA were delivered at 128 d GA.

### Method Details

#### Fetal and Maternal Blood Collection and Delivery

Maternal plasma samples for analysis were taken immediately before operative delivery. Ewes received an intravenous bolus of midazolam (0.5 mg/kg) and ketamine (10 mg/kg) for the deep induction of anaesthesia. A 3 ml injection of 2% (20 mg/ml) lidocaine was given at L6/L7 for spinal analgesia. Fetal venous umbilical cord plasma samples were taken for analysis during delivery. The fetus was then delivered, and the ewe and fetus euthanized under anaesthesia with pentobarbital. All fetal plasma samples were matched with corresponding maternal plasma samples for analysis.

#### Inflammation-Specific Gene Target Selection

RNA targets were selected based on eight known inflammatory markers associated with adverse, inflammation-driven pregnancy complications[12, 14, 28]. These being *IL-1α, IL-8, IL-6, IL-18, IL-1β, TNFα, CD14* and *IL-10*. Primers were designed and validated to amplify cross-exonic regions by using the UCSC Genome Browser in-silico PCR tool (https://genome.ucsc.edu/). See Table 1 for sheep and Table 2 for human gene target primer specifications, respectively. Table 3 illustrates the housekeeping gene target primers for both sheep and human plasma.

**Table 1:** Gene target primers for Reverse Transcription (RT), Pre-amplification (Pre-amp) and qPCR in sheep plasma

Target Gene	Gene ID		Forward Primer SEQ 5'—3'	Reverse Primer SEQ 5'−3'	Amplicon size (bp)
*IL1 A*	*NM_001009808.1*	*RT*	*-*	*TGATTGAGGGCGTCGTTCAGG*	*-*
		*Pre-amp*	*TGGTGACAGCCAATGGCAAGArUTCTGC/3SpC3/*	*TGATTGAGGGCGTCGTTCAGGrATGCAG/3SpC3/*	*208*
		*qPCR*	*ATGACCTGGAAGCCATTGCCA*	*TTGAGGGCGTCGTTCAGGATG*	*139*
*IL1B*	*NM_001009465.2*	*RT*	*-*	*GCACACATGGGCTATCCAGCA*	*-*
		*Pre-amp*	*AAGCTGAGGAGCCGTGCCTArCGAACC/3SpC3/*	*GCACACATGGGCTATCCAGCArCCAGGT/3SpC3/*	*199*
		*qPCR*	*CGTGATGATGACCTGAGGAGCA*	*TTACTGACTGCACGGCTGCAT*	*109*
*IL6*	*NM_001009392.1*	*RT*	*-*	*CATGTCAGTGTGTGTGGCTGGA*	*-*
		*Pre-amp*	*TACCTGGACTTCCTCCAGAACGArGTTTGC/3SpC3/*	*CATGTCAGTGTGTGTGGCTGGArGTGGTC/3SpC3/*	*141*
		*qPCR*	*CTGTCATGGAGTTGCAGAGCAGT*	*GTCAGTGTGTGTGGCTGGAGT*	*95*
*IL8*	*NM_001009401.2*	*RT*	*-*	*TCTGAATTTTCGCAGTGTGGCCC*	*-*
		*Pre-amp*	*AGCTGGCTGTTGCTCTCTTGGrCCGCTC/3SpC3/*	*TCTGAATTTTCGCAGTGTGGCCCrACTCTT/3SpC3/*	*184*
		*qPCR*	*CTGTTGCTCTCTTGGCCGCTT*	*GGGTGGAAAGGTGTGGAATGTGTT*	*121*
*TNFα*	*NM_001024860.1*	*RT*	*-*	*ATGCGGCTGATGGTGTGGGT*	*-*
		*Pre-amp*	*GCCACCACGCTCTTCTGCCTrGCTGCC/3SpC3/*	*ATGCGGCTGATGGTGTGGGTrGAGGAC/3SpC3/*	*347*
		*qPCR*	*GCTCTTCTGCCTGCTGCACTTC*	*CCTGAGTGTCTGAACCAGAGGC*	*100*
*IL18*	*NM_001009263.2*	*RT*	*-*	*TGCACAGAGATGGTTACAGCCAG*	*-*
		*Pre-amp*	*GCCAGGGAAATCAACCTGTCTTTGrAGGATC/3SpC3/*	*TGCACAGAGATGGTTACAGCCAGrACCTCG/3SpC3/*	*133*
		*qPCR*	*GCCAGGGAAATCAACCTGTCTTTG*	*ACAGCCAGACCTCTAGTGAGGC*	*118*
*IL10*	*NM_001009327.1*	*RT*	*-*	*GCCTTGCTCTTGTTTTCGCAGG*	*-*
		*Pre-amp*	*TGGAGGAGGTGATGCCACAGGrCTGAGC/3SpC3/*	*GCCTTGCTCTTGTTTTCGCAGGrGCAGAG/3SpC3/*	*142*
		*qPCR*	*ACAGGCTGAGAACCATGGGC*	*CGCAGGGCAGAAAACGATGACAG*	*110*
*CD14*	*NM_001077209.2*	*RT*	*-*	*CCATACACTGAACGGCGCTA*	*-*
		*Pre-amp*	*GCGTGAGCCACTGTAAAGGArAAGAAT/3SpC3/*	*CCATACACTGAACGGCGCTArGACCAT/3SpC3/*	*242*
		*qPCR*	*ACAGTCCAGCCGACAACCAG*	*ACAGCGGAAATCGTCGTCGT*	*159*

**Table 2:** Gene target primers for Reverse Transcription (RT), Pre-amplification (Pre-amp) and qPCR in human plasma

Target Gene	Gene ID		Forward Primer SEQ 5'—3'	Reverse Primer SEQ 5'−3'	Amplicon size (bp)
*IL1 A*	*NM_000575.5*	*RT*	*-*	*CAGCAGCCGTGAGGTACTGAT*	*-*
		*Pre-amp*	*GCAACCAACGGGAAGGTTCTGrAAGAAT/3SpC3/*	*CAGCAGCCGTGAGGTACTGATrCATTGA/3SpC3/*	*244*
		*qPCR*	*CGGGAAGGTTCTGAAGAAGAGACG*	*AAGGTGCTGACCTAGGCTTGATGA*	*116*
*IL1B*	*NM_000576.3*	*RT*	*-*	*CGGAGCGTGCAGTTCAGTGAT*	*-*
		*Pre-amp*	*AGGCCGCGTCAGTTGTTGTGrGCCATA/3SpC3/*	*CGGAGCGTGCAGTTCAGTGATrCGTACC/3SpC3/*	*193*
		*qPCR*	*GAGGAAGATGCTGGTTCCCTGC*	*AGCCTCGTTATCCCATGTGTCGAA*	*115*
*IL6*	*NM_000600.5*	*RT*	*-*	*TTCTGTGCCTGCAGCTTCGT*	*-*
		*Pre-amp*	*AACAAGCCAGAGCTGTGCAGATGrAGTACC/3SpC3/*	*TTCTGTGCCTGCAGCTTCGTrCAGCAT/3SpC3/*	*136*
		*qPCR*	*CCAGAGCTGTGCAGATGAGTACAAA*	*GCAGGCTGGCATTTGTGGTTG*	*108*
*IL8*	*NM_000584.4*	*RT*	*-*	*GTGTTGGCGCAGTGTGGTC*	*-*
		*Pre-amp*	*CAGCTCTGTGTGAAGGTGCAGTTrUTGCCG/3SpC3/*	*GTGTTGGCGCAGTGTGGTCrCACTCG/3SpC3/*	*142*
		*qPCR*	*AGCTCTGTGTGAAGGTGCAGTT*	*TGTTGGCGCAGTGTGGTCC*	*140*
*TNFα*	*NM_000594.4*	*RT*	*-*	*ATGCGGCTGATGGTGTGGGT*	*-*
		*Pre-amp*	*GCCACCACGCTCTTCTGCCTrGCTGCC/3SpC3/*	*ATGCGGCTGATGGTGTGGGTrGAGGAC/3SpC3/*	*347*
		*qPCR*	*TGCTGCACTTTGGAGTGATCGG*	*GCTACAACATGGGCTACAGGCT*	*133*
*IL18*	*NM_001562.4*	*RT*	*-*	*GCCATACCTCTAGGCTGGCTATCT*	*-*
		*Pre-amp*	*CATTGACCAAGGAAATCGGCCTCrUATTTA/3SpC3/*	*GCCATACCTCTAGGCTGGCTATCTrUTATAT/3SpC3/*	*120*
		*qPCR*	*TTGACCAAGGAAATCGGCCTCT*	*GCCATACCTCTAGGCTGGCTAT*	*118*
*IL10*	*NM_000572.3*	*RT*	*-*	*AAGGCATTCTTCACCTGCTCCAC*	*-*
		*Pre-amp*	*CCCAGACATCAAGGCGCATGTrGAACTA/3SpC3/*	*AAGGCATTCTTCACCTGCTCCACrGGCCTC/3SpC3/*	*132*
		*qPCR*	*TCAAGGCGCATGTGAACTCCC*	*AGGCATTCTTCACCTGCTCCAC*	*123*
*CD14*	*NM_000591.4*	*RT*	*-*	*TCCGCATCGACGCGCTTTA*	*-*
		*Pre-amp*	*GCACTTCCAGAGCCTGTCCGrGAGCTT/3SpC3/*	*TCCGCATCGACGCGCTTTArGAAACT/3SpC3/*	*277*
		*qPCR*	*CGGAAGACTTATCGACCATGGAGC*	*GGACCAGTCGGGCTGAGGTT*	*155*

**Table 3:** Housekeeping gene target primers for Reverse Transcription (RT), Pre-amplification (Pre-amp) and qPCR in sheep and human plasma

Target Gene	Gene ID		Forward Primer SEQ 5'—3'	Reverse Primer SEQ 5'−3'	Amplicon size (bp)
ACTB	NM_001009784.3 (Sheep)	RT	-	ATG GCA GGG GTG TTG AAG	-
		Pre-amp	ACACCTTCTACAACGAGCTGCrGTGTGA/3SpC3/	ATGGCAGGGGTGTTGAAGrGTCTCT/3SpC3/	133
		qPCR	ACACCTTCTACAACGAGCTGC	ATGGCAGGGGTGTTGAAG	133
RPS18	XM_004018745.5 (Sheep)	RT	-	GTGGGCCCGAATCTTCTTCA	-
		Pre-amp	GGAACGTGTGATCACCATTATGCrAGAATT/3SpC3/	GTGGGCCCGAATCTTCTTCArGACGCA/3SpC3/	169
		qPCR	GGAACGTGTGATCACCATTATGC	GTGGGCCCGAATCTTCTTCA	169
GAPDH	NM_001190390.1 (Sheep)	RT	-	CAAACATGGGAGCGTCAGC	-
		Pre-amp	TGGAGTCCACTGGGGTCTTCrACTACT/3SpC3/	CAAACATGGGAGCGTCAGCrAGAAGA/3SpC3/	108
		qPCR	TGGAGTCCACTGGGGTCTTC	CAAACATGGGAGCGTCAGC	108
LUC		RT	-	GCGGTCGGTAAAGTTGTT	-
		Pre-amp	CTCTGATTAACGCCCAGCrGTTTTG/3SpC3/	GCGGTCGGTAAAGTTGTTrCCATTA/3SpC3/	
		qPCR	CTCTGATTAACGCCCAGC	GCGGTCGGTAAAGTTGTT	
ACTB	NM_001101.5 (Human)	RT	-	TGGAGTTGAAGGTAGTTTCG	-
		Pre-amp	AGAAGAGCTACGAGCTGCCTGArCGGCCC/3SpC3/	TGGAGTTGAAGGTAGTTTCGrUGGATT/3SpC3/	135
		qPCR	CATCACCATTGGCAATGA	AAGGTAGTTTCGTGGATGC	96
RPS18	NM_022551.3 (Human)	RT	-	AAGAACCAGTCTGGGATCTT	-
		Pre-amp	TATGCTCATGTGGTGTTGAGrGAAAGG/3SpC3/	AAGAACCAGTCTGGGATCTTrGTACTC/3SpC3/	134
		qPCR	AGACATTGACCTCACCAAGA	TTGTACTGGCGTGGATTC	90
GAPDH	NM_002046.7 (Human)	RT	-	CATGAGTCCTTCCACGATA	-
		Pre-amp	ATGTTCGTCATGGGTGTGrAACCAG/3SpC3/	CATGAGTCCTTCCACGATArCCAAAC/3SpC3/	138
		qPCR	AGAAGTATGACAACAGCCTCA	CCAAAGTTGTCATGGATGA	94

#### Target Primer Design

RNase H-dependent PCR (rhPCR) primers were designed according to Integrated DNA Technologies Gen1 design. The primer consists of five different parts starting at the 5’ end with the final functional primer (comprising of more than 10 DNA bases that matches the template), the cleavage site (single RNA residue), four matching DNA bases, one mismatch DNA base, and lastly the blocking group (C3 spacer) at the 3’ end [29, 30].

#### Circulating RNA Extraction

cfRNA was extracted from 250 μl of maternal plasma using a Plasma/Serum Circulating and Exosomal RNA Purification Kit (Norgen, Ontario, Canada; Cat no. 42800). The residual DNA in the cfRNA was digested using a RNase-Free DNase I Kit (Norgen, Cat no. 25720). Extracted cfRNA was purified using a RNA Clean and Concentrator™−5 kit (Zymo, Irvine, CA; Cat no. ZYR. R1016), yielding 20 μL of cfRNA per sample.

#### Reverse Transcription and Emulsion Based Targeted Pre-amplification

10 μL of extracted cfRNA was annealed with a final concentration of 0.4 μM of pooled reverse primer mix in the presence of 10^5^ copies of luciferase control RNA (Promega, Cat no. L4561) and a final concentration 2 mM of dNTPs at 65 °C for 5 min. Reverse transcription of cfRNA was performed using Superscript™ III Reverse Transcriptase (Thermo Fisher Scientific, Waltham, MA; Cat no. 18080044) at 25 °C for 5 min, 50 °C for 50 min, and enzyme inactivation at 95 °C for 3 min.

cDNA from reverse transcription was added to a PCR mixture comprised of Platinum™ Taq DNA Polymerase (Thermo Fisher Scientific; Cat no. 10966) with a final concentration of 0.5 μM of rh PCR primer mix and 26 mU of RNase H2 enzyme. An emulsion was generated by adding three parts of 10% 008-FluoroSurfactant (RAN Biotechnologies, Beverley, MA) in 3 M Fluorinert ™ Engineered Fluid (3 M, Saint Paul, MN; Cat no. FC-40) to one part of PCR reaction mixture. The mixture was shaken using a sharp flicking motion for 30 s, until it became cloudy and uniform. Thermocycling started with enzyme activation at 94 °C for 2 min, followed by 20 cycles of denaturation (94 °C, 15 s), annealing (55 °C, 30 s), and extension (68 °C, 1 min). Reaction mixtures were frozen overnight at −75 °C to release PCR products from emulsions. Samples were defrosted at room temperature and the aqueous phase removed. Reactions recovered from the emulsion PCR was topped up with the same amount of polymerase and RNase H2 used in the emulsion PCR. Thermocycling commenced with enzyme activation at 94 °C for 2 min, followed by 20 cycles of denaturation (94 °C, 15 s), hybridization (78 °C, 10 min), annealing (55 °C, 30 s), and extension (68 °C, 1 min).

#### Quantification of Pre-amplified Gene Targets Using qPCR

qPCR was performed for 60 cycles with Maxima SYBR Green/ROX qPCR Master Mix (Thermo Fisher, Cat no. K0221). 1 μl of the amplified target, 0.3 μM of the forward and reverse primers are added to 12.5 μl of the master mix. Water was then added to form a total 25 μl reaction volume. The mix was then run using a 96 well QuantStudio™ 3 quantitative PCR cycler (Thermo Fisher Scientific) using the following thermal profile: 95 °C for 10 min for the initial denaturation, followed by 60 cycles of 95 °C denaturation (15 s) and 65 °C annealing and extension (60 s). SYBR Green/ROX Quantification was performed at the end of each extension cycle. Melt curve analysis was performed by ramping the temperature to 95 °C (0.3 °C/second) after the qPCR run completed to verify the specificity of each PCR product.

#### Placental Tissue mRNA extraction

RNA was extracted from frozen placental tissue using a Precellys Evolution Cryolys (Bertin Technologies, Ile-de-France, France) programmed as follows: single cycle, 6000 rpm, 5 s, 4 °C. Tissue lysates were processed using an RNeasy Plus Mini Kit (Qiagen, Hilden, Germany) according to the manufacturer’s instructions. The concentration and integrity of extracted RNA was determined using an RNA 6000 Nano Kit and measured on a 2100 Bioanalyser system (both Agilent Technologies, Inc., Santa Clara, CA). All RNA extracts had an RIN value > 8 and were diluted in nuclease-free water (Life Technologies) to yield a final RNA concentration of 25 ng/μL.

qPCR was performed using cycles with Maxima SYBR Green/ROX qPCR Master Mix (Thermo Fisher, Cat no. K0221). 1 μl of the amplified target, 0.3 μM of the forward and reverse primers are added to 12.5 μl of the master mix. Water was then added to form a total 25 μl reaction volume. The mix was then run using a 96 well QuantStudio™ 3 quantitative PCR cycler (Thermo Fisher Scientific) using the following thermal profile: 95 °C for 10 min for the initial denaturation, followed by 60 cycles of 95 °C denaturation (15 s) and 65 °C annealing and extension (60 s). SYBR Green/ROX Quantification was performed at the end of each extension cycle. Melt curve analysis was performed by ramping the temperature to 95 °C (0.3 °C/second) after the qPCR run completed to verify the specificity of each PCR product fetal lung mRNA transcripts corresponding to the top 5 RNA targets in maternal and fetal plasma were measured, using the same primers and SYBR chemistry with identical cycling parameters. Delta quantification cycle values were used to determine relative expression of transcripts for statistical analyses. Final data were expressed graphically as fold increase compared to Saline Control Group value.

#### Human Plasma Samples from Patients with Late Onset Pre-Eclampsia

Samples from pregnancies complicated by PE and from normotensive controls were collected at the Gynecology and Obstetrics Department of Hospital Parroquial de San Bernardo, Santiago, Chile. All women enrolled in this study gave written informed consent for the collection of samples and information. This research was approved by the Ethical Scientific Committees of Universidad de los Andes, Chile. Cases included women with a singleton pregnancy who subsequently developed PE, and the controls included women with a singleton pregnancy without chronic medical conditions or obstetric complications. PE was defined as late-onset PE with an onset of clinical signs and symptoms and delivery after 34 weeks of gestation. Blood samples were collected at 11–13 (early), 22–24 (mid) and 32–34 (late) weeks in citrate BD Vacutainer tubes (BD Biosciences, San Jose, CA, USA), followed by centrifugation at 1500 × *g* for 15 min. Plasma fractions were separated and aliquots were stored at − 80 °C until further analysis.

### Quantification and Statistical Analysis

Statistical analysis was performed using IBM SPSS Statistics for Windows Version 28.0.1.0. All numerical data were tested for normality with Shapiro–Wilk tests. In the comparison of the intervention groups (Saline Control Groups, 2 d LPS, 8 d LPS) between-group differences in parametric data were tested for significance with one-way ANOVA, while Kruskal Wallis tests were used for non-parametric data. Multiple *post-hoc* comparisons were performed with Tukey’s and Dunnett t (2-sided) tests, p values < 0.05 being significant. For the analysis of the fetal haematological data and placental qPCR targets between the Infectious Chorioamnionitis and Media control groups at two different gestations, two sample t-test was utilized for parametric data, and Mann Whitney U Test for non-parametric data, p values < 0.05 being significant.

### Data Analysis

Cycle threshold (Ct) values of the transcripts underwent two normalization steps. The first normalization step was to normalize to the Ct value of the luciferase calibrator (spiked into the sample prior to reverse transcription and emulsion amplification), which accounted for technical variation during the PCR steps across different samples. The second normalization step followed standard delta-delta-Ct normalisation using the geometric mean of the three reference genes (GAPDH, RPS18, Beta Actin) and accounted for variation in the amount of extracted RNA. Normalized Ct values for each target were used as features for classification. Random Forest algorithm from the Random Forest library in R was used to identify genes that separate groups demonstrating an upregulation in inflammatory markers compared to those not[31, 32]. We further employed the Random Forest method to determine the area under the receiver operating characteristic curve (AUC) for the feature sets[33]. Different combinations of the same eight gene targets consisting of maternal Cts at baseline compared to delivery (post control or inflammatory intervention) were explored as feature sets. Combinations of 4 of the 8 targets (*IL-1α*, *IL-1β*, *IL6*, *CD14* compared to *IL8*, *IL10*, *IL18*, *TNFα*) in each of the maternal feature sets were further explored in isolation to investigate whether this improved the diagnostic accuracy. AUC was used as the metric to compare the validity of each feature in distinguishing inflammation from no inflammation. Raw Ct data and related R Scripts are contained within the Supplementary Data.

## Results

### A Sterile Intrauterine Inflammatory Insult Was Detectable By Fetal, but not Maternal Differential Blood Cell Analyses at 8 Days Post Intrauterine LPS Exposure

**Fig. 1:**
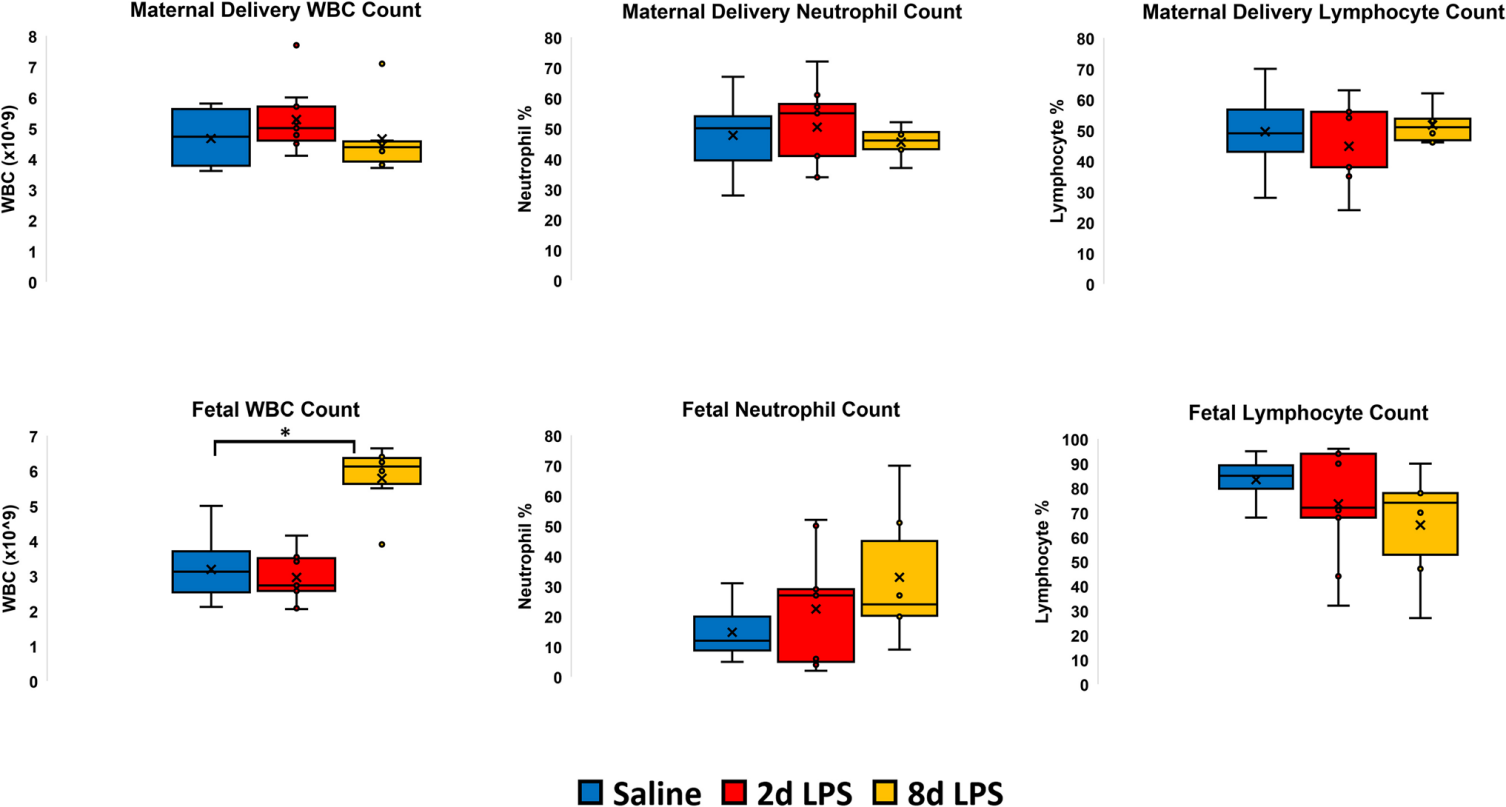
Maternal and fetal haematological data showing White Blood Cell count (WBC), Neutrophil and Lymphocyte counts at delivery for Saline control, 2 Day LPS and 8 Day LPS exposed groups. The Asterix indicates a significant difference among the groups. Error bars represent ± 1 Standard Deviation

Total maternal and fetal white blood cell (WBC), neutrophil and lymphocyte counts at delivery are shown in Fig. 1. There was no statistically significant difference in maternal total WBC, neutrophil and lymphocyte counts at delivery between the Negative Control Group and the 2 d or 8 d Sterile Chorioamnionitis Groups. There were statistically significant increases in total WBC counts (p < 0.001) between lambs from the Negative Control Group and the 8 d Sterile Chorioamnionitis Group, consistent with intrauterine exposure to endotoxin. Fetal WBC counts are not routinely available in the clinical assessment of chorioamnionitis.

### The Placental Inflammatory Response to Sterile Intrauterine LPS At 2 and 8 Days of Exposure

**Fig. 2:**
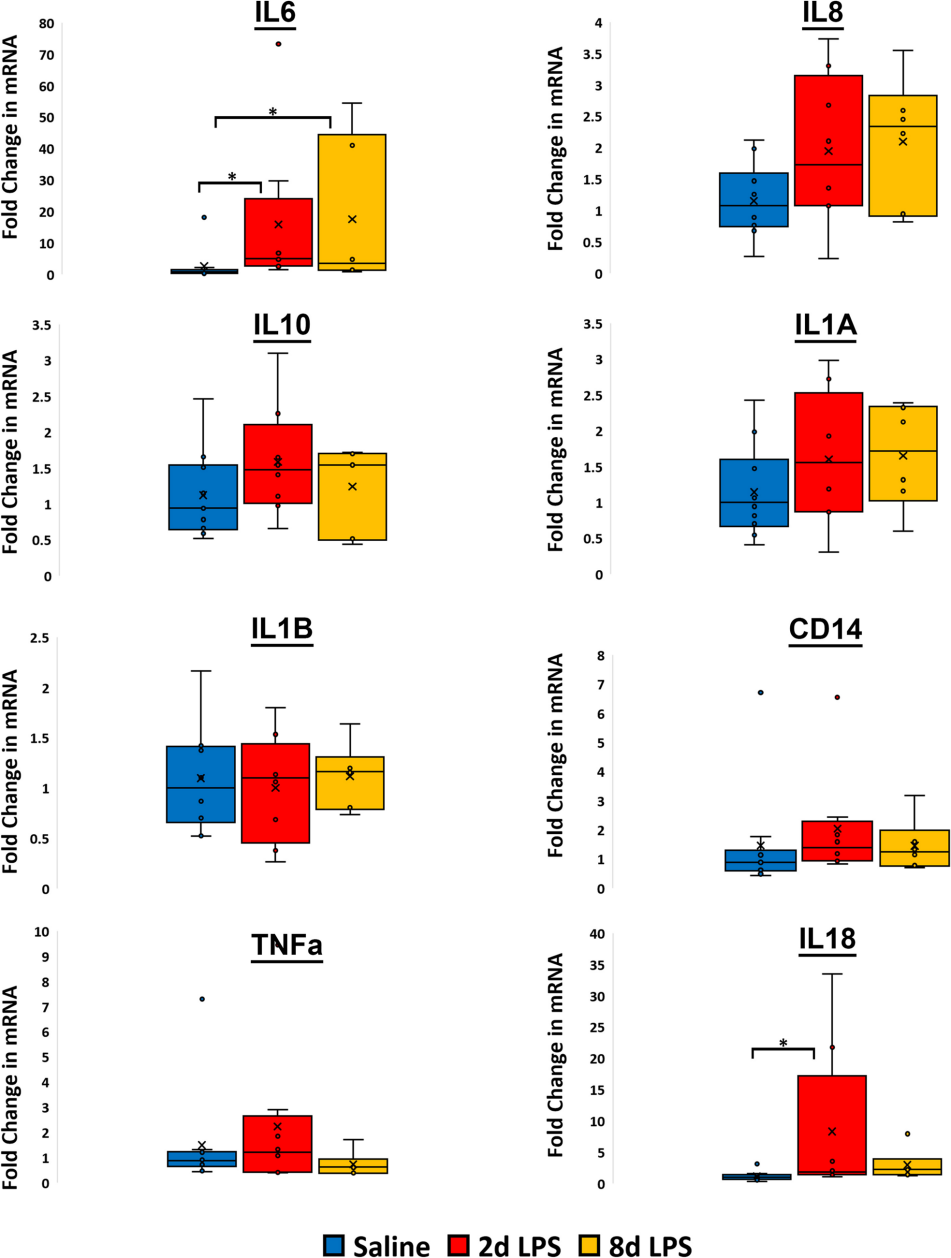
mRNA quantification from placental tissue in fold change values of inflammatory targets exposed to LPS at 2 days and 8 days relative to Saline control. Relative mRNA expression of mRNA transcripts for the intra-uterine inflammatory gene targets *IL6, IL8, IL10, IL1α, IL1β, CD14, TNFα, IL18.* The Asterix indicates a significant difference among the groups. Error bars represent ± 1 Standard Deviation

mRNA was extracted from placental samples to assess the placental inflammatory response to sterile intrauterine LPS exposure. Placental mRNA expression of inflammatory targets was measured using qPCR for each intervention group. Cycle threshold (Ct) values for each individual mRNA inflammatory target for the Negative Control and Sterile Chorioamnionitis Groups (both 2 d and 8 d LPS exposure) from the placenta are shown as fold changes in Fig. 2. Compared to Negative Control Group animals, the 2 d Sterile Chorioamnionitis Group animals had significantly increased placental mRNA for *IL6* (mean dCt difference = 2.77; 95% CI 5.01 to 0.0.53, p = 0.015) and *IL18* (mean dCt difference = 1.79; 95% CI 3.28 to 0.29, p = 0.019). Animals from the 8 d Sterile Chorioamnionitis Group also had significantly increased placental mRNA for *IL6* (mean dCt difference = 2.51; 95% CI 4.95 to 0.07, p = 0.044) compared to the Negative Control Group. These findings demonstrate measurable placental inflammatory response to LPS at 2 and 8 days after exposure providing further evidence of a robust model of sterile chorioamnionitis.

### A Targeted Cell-Free RNA Analyses of Maternal Plasma Is Predictive of Sterile Intrauterine Inflammation Modelling Chorioamnionitis

**Fig. 3:**
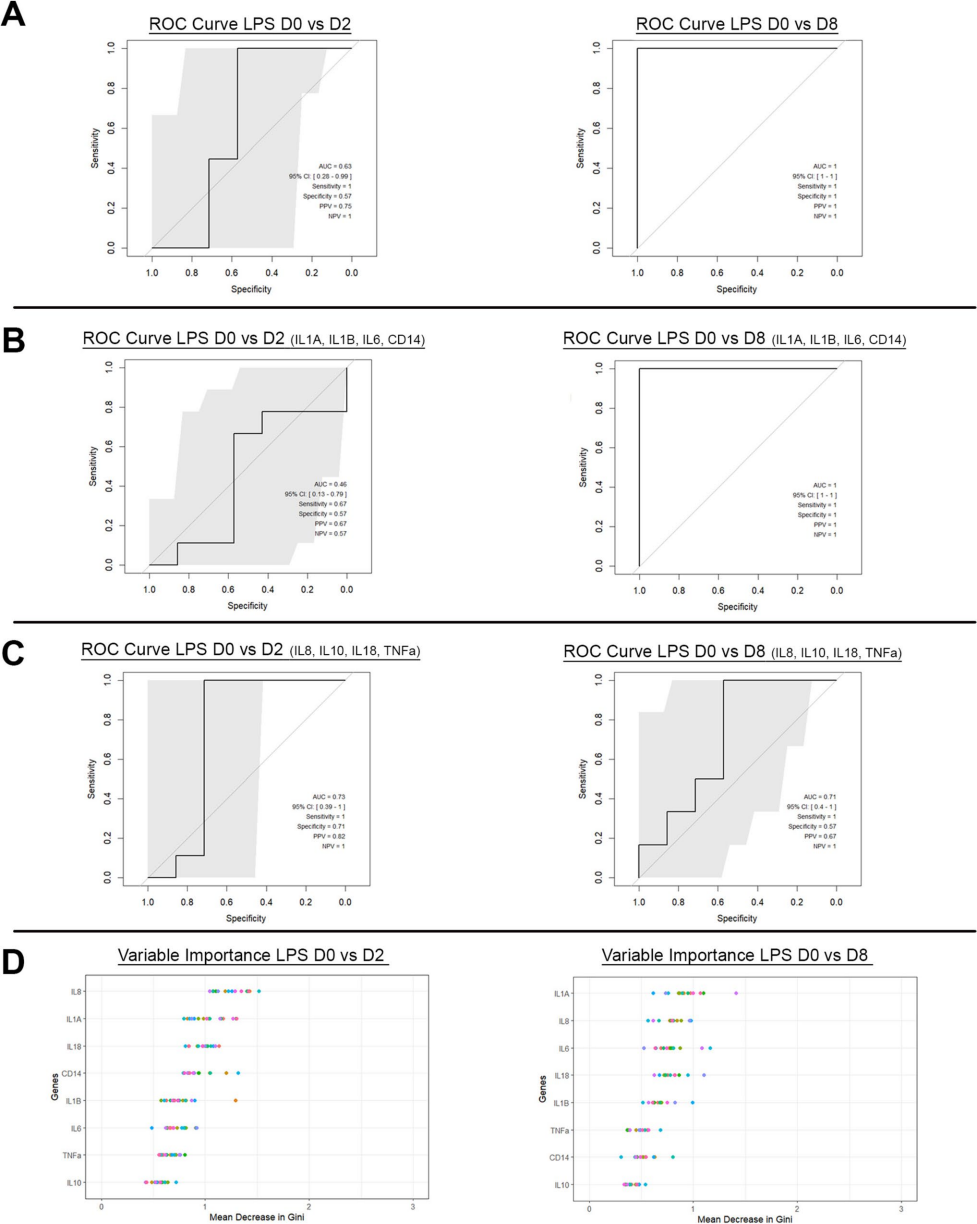
**A** Area Under the Curve Plot for maternal sheep plasma inflammatory cfRNA signal for 2- and 8-d LPS exposed groups. **B** Area Under the Curve Plot for maternal sheep plasma inflammatory cfRNA signal for 2- and 8-d LPS exposed group partial panel targets (*IL1α, IL1β, IL6, CD14*). **C** Area Under the Curve Plot for maternal sheep plasma inflammatory cfRNA signal for 2- and 8-day LPS exposed group partial panel Targets (*IL8, IL10, IL18, TNFα*). **D** GINI plots for 2 and 8 days LPS exposure demonstrating the order of genes of variable importance

Maternal standardized Ct values for each individual cfRNA target are shown in the Supplementary Data. Random Forrest Classifier plots for maternal plasma gene target Cts are shown in Fig. 3A for 2 d and 8 d Sterile Chorioamnionitis Group animals. Analyses were performed using a panel of eight cfRNA targets related to inflammation (*IL1 A, IL1β, IL6, IL8, IL10, IL18, CD14, TNFα)*. Targets were chosen based on their strong link to adverse inflammatory pathology in pregnancy, and with adverse newborn outcomes[34]. Maternal plasma Ct feature sets after 2 d of LPS exposure had mean AUC of 0.63 (95% CI 0.28–0.99), Sensitivity 100%, Specificity 57%, Positive Predictive Value (PPV) 0.75, Negative Predictive Value (NPV) 1.0 (Fig. 3A and Table 4). In comparison, maternal plasma Ct feature sets after 8 d LPS exposure had mean AUC of 1.0 (95% CI 1–1), Sensitivity 100%, Specificity 100%, PPV 1.0, NPV 1.0 (Fig. 3A and Table 4). In order to assess if a reduced number of cfRNA targets increased predictive power in the 2 d LPS group, subsequent analyses were performed on 2 different subgroups, each with 4 targets. Targeted analysis of *IL1 A, IL1B, IL6,* and *CD14* in maternal plasma of the 2 d Sterile Chorioamnionitis Group animals did not improve predictive values with a mean AUC of 0.46 (95% CI 0.13–0.79), Sensitivity 67%, Specificity 57%, PPV 0.67, NPV 0.57 (Fig. 3B, Table 4). Whilst the same targets demonstrated a mean AUC of 1.0 (95% CI 1–1) in the 8 d Sterile Chorioamnionitis Group animals indicating that these four targets are critical to the predictive value at 8 days of LPS exposure. (Fig. 3B, Table 4).

**Table 4:** Summary of the predictive accuracy of targeted cfRNA expression in maternal plasma with different inflammatory exposures

	**AUC [95% CI]**	**Sensitivity**	**Specificity**	**PPV**	**NPV**
Sheep 2 d LPS (Entire Panel)	0.63 [0.28 −0.99]	1	0.57	0.75	1
Sheep 2 d LPS (*IL8, IL10, IL18, TNFα*)	0.73 [0.39–1]	1	0.71	0.82	1
Sheep 2 d LPS (*IL1α, IL1β, IL6, CD14*)	0.46 [0.13–0.79]	0.67	0.57	0.67	0.57
Sheep 8 d LPS (Entire Panel)	1.0 [1-1]	1	1	1	1
Sheep 8 d LPS (*IL8, IL10, IL18, TNFα*)	0.71 [0.4–1]	1	0.57	0.67	1
Sheep 8 d LPS (*IL1α, IL1β, IL6, CD14*)	1.0 [1-1]	1	1	1	1
Sheep 98 d *Mycoplasma hominis*	0.84 [0.6–1]	0.62	1	1	0.57
Sheep 128 d *Mycoplasma hominis*	0.73 [0.46–1]	0.75	0.75	0.75	0.75
Human 1 st Trimester PE	0.85 [0.65–1]	0.9	0.9	0.9	0.9
Human 2nd Trimester PE	0.90 [0.75–1]	1	0.8	0.83	1
Human 3rd Trimester PE	0.82 [0.62–1]	0.8	0.8	0.8	0.8

*PE* pre-eclampsia

In contrast, targeted analysis of *IL8, IL10, IL18, TNFα* in maternal plasma of the 2 d Sterile Chorioamnionitis Group animals improved predictive value with a mean AUC of 0.73 (95% CI 0.39–1) Sensitivity 100%, Specificity 71%, PPV 0.82, NPV 1.0 (Fig. 3C, Table 4). The same targets in the 8 d Sterile Chorioamnionitis Group animals had a mean AUC of 0.71 (95% CI 0.4–1) (Fig. 3C, Table 4). The relative contribution of each cfRNA gene target to the overall predictive power of detecting chorioamnionitis in the maternal plasma is shown in Fig. 3D for both the 2 d and 8 d Sterile Chorioamnionitis Groups. *IL8* and *IL1 A* were the two most important targets at 2 d and 8 d of LPS exposure, contributing the most variable importance for accurate prediction of intrauterine inflammation. These findings demonstrate that targeted analysis of a cell-free RNA signature in maternal plasma serves as an accurate predictor of sterile intrauterine inflammation.

### The Fetal Haematological Inflammatory Response to an Infectious Intrauterine Exposure (Mycoplasma hominis)

Microbial inflammatory insults (as opposed to a standardised endotoxin exposure) are more variable in terms of the magnitude of inflammatory response generated and the range of inflammatory pathways activated. In order to model an infectious intrauterine exposure and assess inflammation we utilised the same sheep model of pregnancy with a singleton fetus at either 91 d GA (extremely early preterm gestation equivalent to ~ 25 weeks’ human gestation) or 121 d GA (early preterm gestation equivalent to ~ 32 weeks’ human gestation). Ewes received an ultrasound guided intra-amniotic injection of either: **i)** Sterile *Mycoplasma hominis* culture broth (Media Negative Control Group; n = 4 per gestational age group); or **ii)** 2 × 10^7^ colour change units (CCU) of *Mycoplasma hominis* bacteria (Infectious Chorioamnionitis Group; *n* = 8 per gestational age group). Surgical delivery of the fetus occurred 7 days post exposure. Animals exposed to either sterile media or *Mycoplasma hominis* at 91 d GA were delivered at 98 d GA. Animals exposed to either sterile media or *Mycoplasma hominis* at 121 d GA were delivered at 128 d GA.

**Fig. 4:**
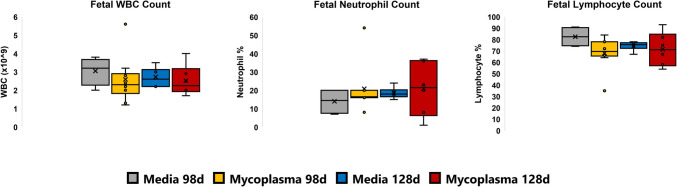
Fetal haematological data showing White Blood Cell count (WBC), Neutrophil and Lymphocyte counts at delivery for Media Negative Control and *Mycoplasma hominis* Infectious Chorioamnionitis Groups at 98 d GA and 128 d GA. The Asterix indicates a significant difference among the groups. Error bars represent ± 1 Standard Deviation

Fetal WBC, neutrophil and lymphocyte counts at delivery for the Media Negative control and Infectious Chorioamnionitis Groups at 98 d GA and 128 d GA are illustrated in Fig. 4. There was no statistically significant difference between groups at the same gestational age of delivery. We hypothesise that this finding may be due to variable intrauterine colonisation of the live bacterium in this model as we did not confirm growth equivalence or presence of viable *Mycoplasma hominis* by culture or qPCR in these delivery samples.

### The Placental Inflammatory Response to an Infectious Intrauterine Exposure (Mycoplasma hominis)

**Fig. 5:**
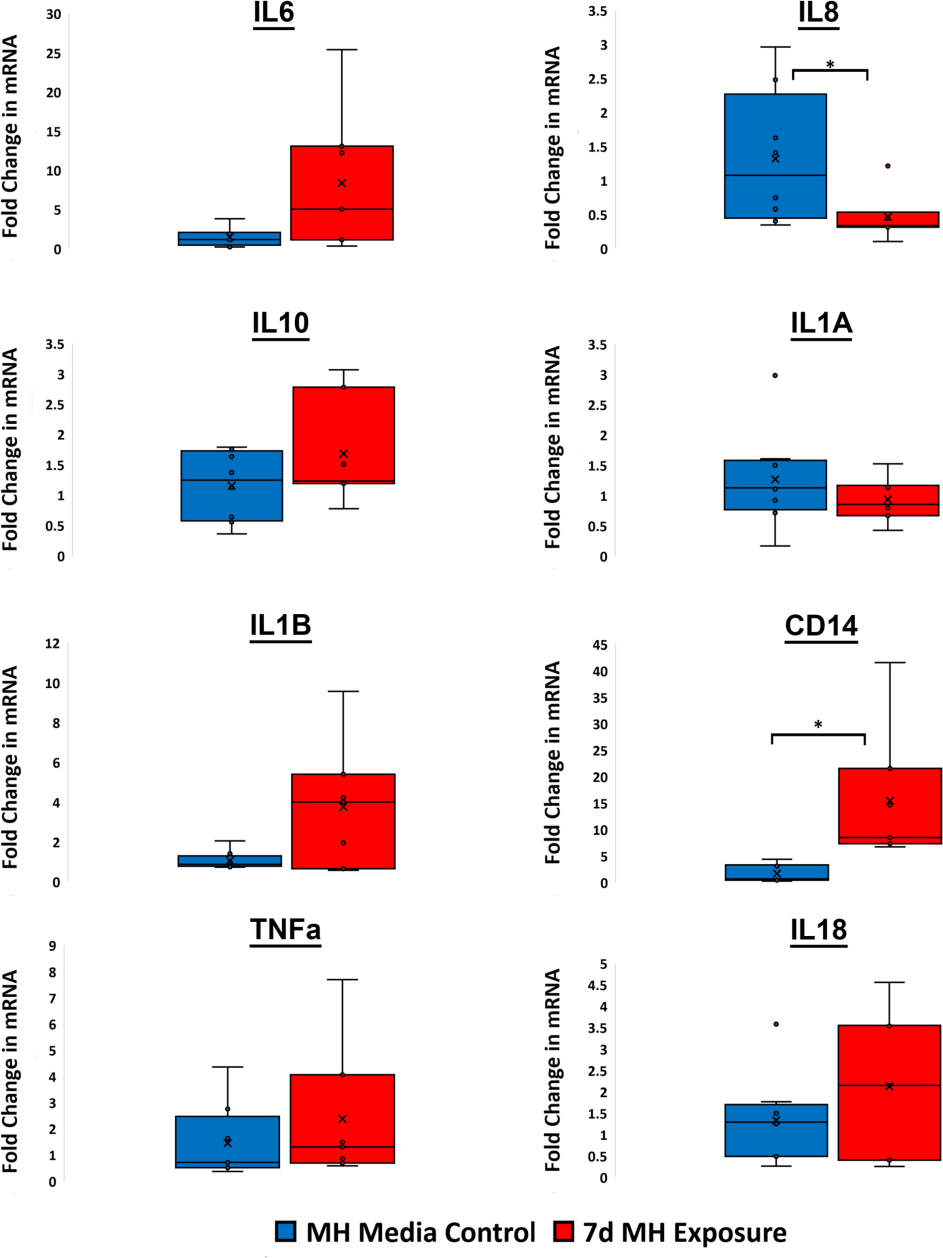
The mRNA quantification from placental tissue in fold change values of inflammatory targets exposed to 7 days of culture media (Control) or *Mycoplasma hominis* at 126 d Gestation. Relative mRNA expression of mRNA transcripts for the intra-uterine inflammatory gene targets *IL6, IL8, IL10, IL1α, IL1β, CD14, TNFα, IL18.* The Asterix indicates a significant difference among the groups. Error bars represent ± 1 Standard Deviation

mRNA expression of a panel of inflammatory targets was measured using qPCR to assess the placental inflammatory response to 7 days of intrauterine *Mycoplasma hominis* exposure, relative to control. Placental Ct values for each individual mRNA inflammatory target for 128 d gestation Media Control group and Infectious Chorioamnionitis Group animals are demonstrated as fold changes in Fig. 5. Infectious Chorioamnionitis Group animals had a statistically significant increase in the expression of CD14 (mean difference = 3.62; 95% CI 2.1 to 5.1, p < 0.001) and significantly decreased expression of IL8 (mean difference = −1.43; 95% CI −2.7 to −0.16, p = 0.03) in comparison to Media Control Group animals (Fig. 5).

### A Targeted cfRNA Signature in Maternal Blood Is Predictive of an Infectious (Mycoplasma hominis) Intra-uterine Exposure

**Fig. 6:**
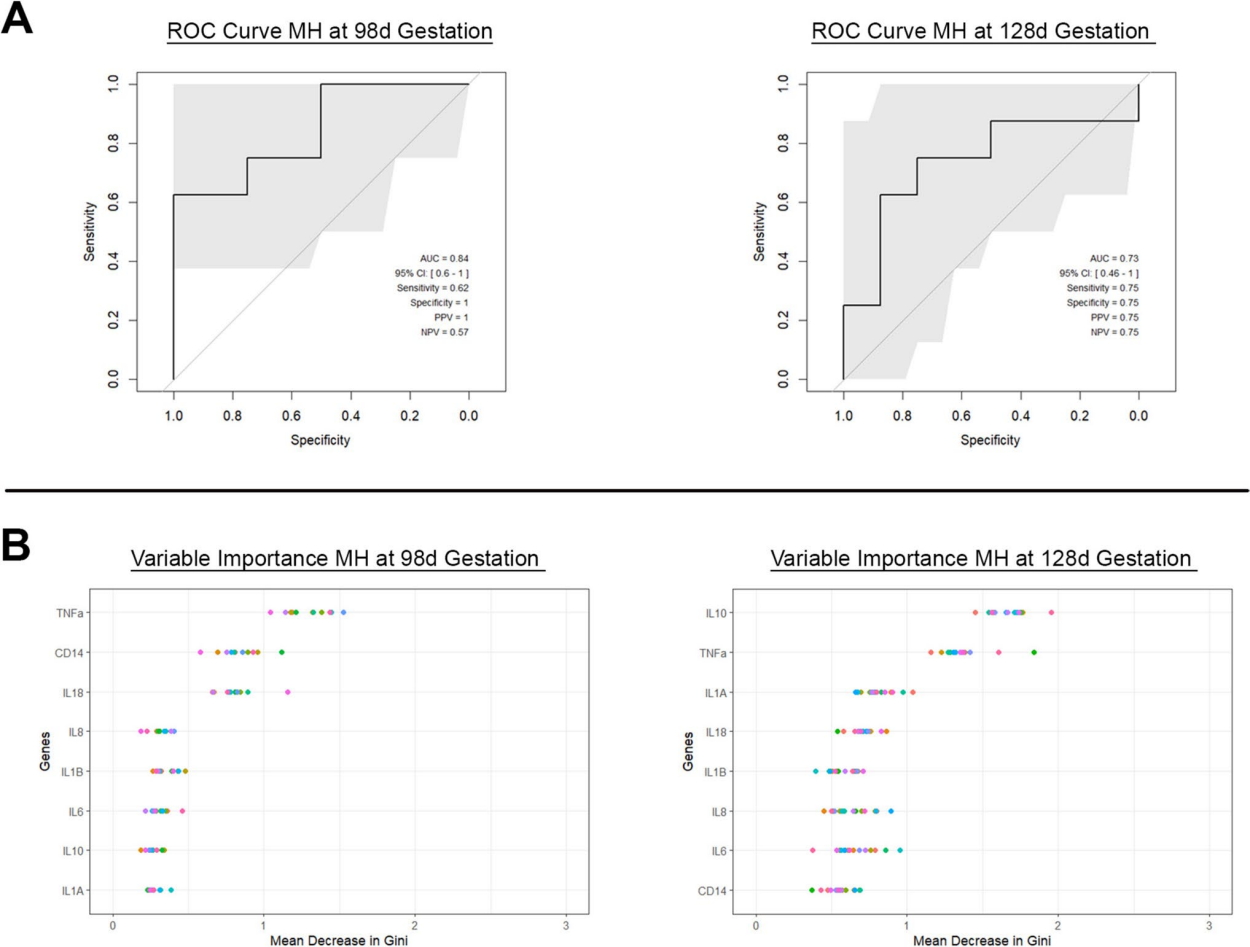
**A** AUC for *Mycoplasma hominis* exposure maternal plasma cfRNA at 98 day gestation and 128 day gestation. **B** GINI plots for 96 day and 126 day gestation *Mycoplasma hominis* exposed groups demonstrating the order of genes of variable importance

We assessed the ability of a targeted analysis of cfRNA expression in maternal plasma to detect an inflammatory response to an infectious bacterial intrauterine exposure (*Mycoplasma hominis*). cfRNA data from maternal plasma from animals exposed to MH Media as well as baseline and post 7 days of intrauterine *Mycoplasma hominis* exposure was analysed with Random Forest Algorithm. Two different gestational ages (98 d and 128 d) were utilised to model extreme prematurity and early prematurity, respectively, informing our understanding of how differences in placental and fetal development may impact the cfRNA. Maternal standardized Ct values for each individual cfRNA target are shown in the Supplementary Data. Random Forrest Classifier plots for maternal plasma gene target Cts are shown in Fig. 6A. Analyses were performed on the same panel of eight cfRNA targets utilised in the Sterile Chorioamnionitis Groups (*IL1α, IL1B, IL6, IL8, IL10, IL18, CD14, TNFα)*. Maternal plasma Ct feature sets at 98 d gestation after *M. hominis* exposure had mean AUC of 0.84 (95% CI 0.6–1), compared to maternal plasma Ct feature sets at 128 d gestation which had mean AUC of 0.73 (95% CI 0.46–1) Fig. 6A, Table 4. The relative contribution of each cfRNA target to the overall model at 98 d and 128 d gestation after *M. hominis* exposure is demonstrated in Fig. 6B. *TNFα* and *CD14* were the two most important targets at 98 d gestation. *IL10* and *TNFα* at were the most important targets at 128 d gestation (Fig. 6B).

### A Targeted cfRNA Signature in Maternal Blood Is Predictive of Pre-Eclampsia in a Human Pregnancy Cohort

The role of inflammation in the pathophysiology of PE is well established. In order to explore the potential utility of our cfRNA approach in screening for PE risk, as well as to technically validate our assays in human samples, we performed plasma analyses collected from a cohort of late onset PE patients and compared these to samples collected from age-matched uncomplicated, term pregnancies. The patient demographics for the PE and control group (uncomplicated term) pregnancies are summarized in Table 5.

**Table 5:** Human plasma sample patient demographics

	**Control (*n* = 10)**	**Pre-Eclampsia (*n* = 10)**		
	**Mean (SD)**	**Mean (SD)**	**P value**	**Statistical analysis**
**Maternal Age (y)**	24.4 ± 6.5	26.9 ± 9.0	0.48	t test
**BMI at 11–14 weeks (kg/m2)**	28.3 ± 7.8	32.6 ± 8.3	0.28	t test
**Parity**	0.8 ± 1	0.7 ± 0.9	0.98	Mann Whitney
**Gravida**	2.3 ± 1.5	1.9 ± 1.2	0.64	Mann Whitney
**Fasting Plasma Glucose (mg/dL)**	72.7 ± 11.7	71.5 ± 9.5	0.81	t test
**Smoker**	1/10	1/10	-	
**Illicit Drug use**	0	0	-	
**Gestational Age at delivery**	39.6 ± 0.8	37.7 ± 1.7	0.006	t test

**Fig. 7:**
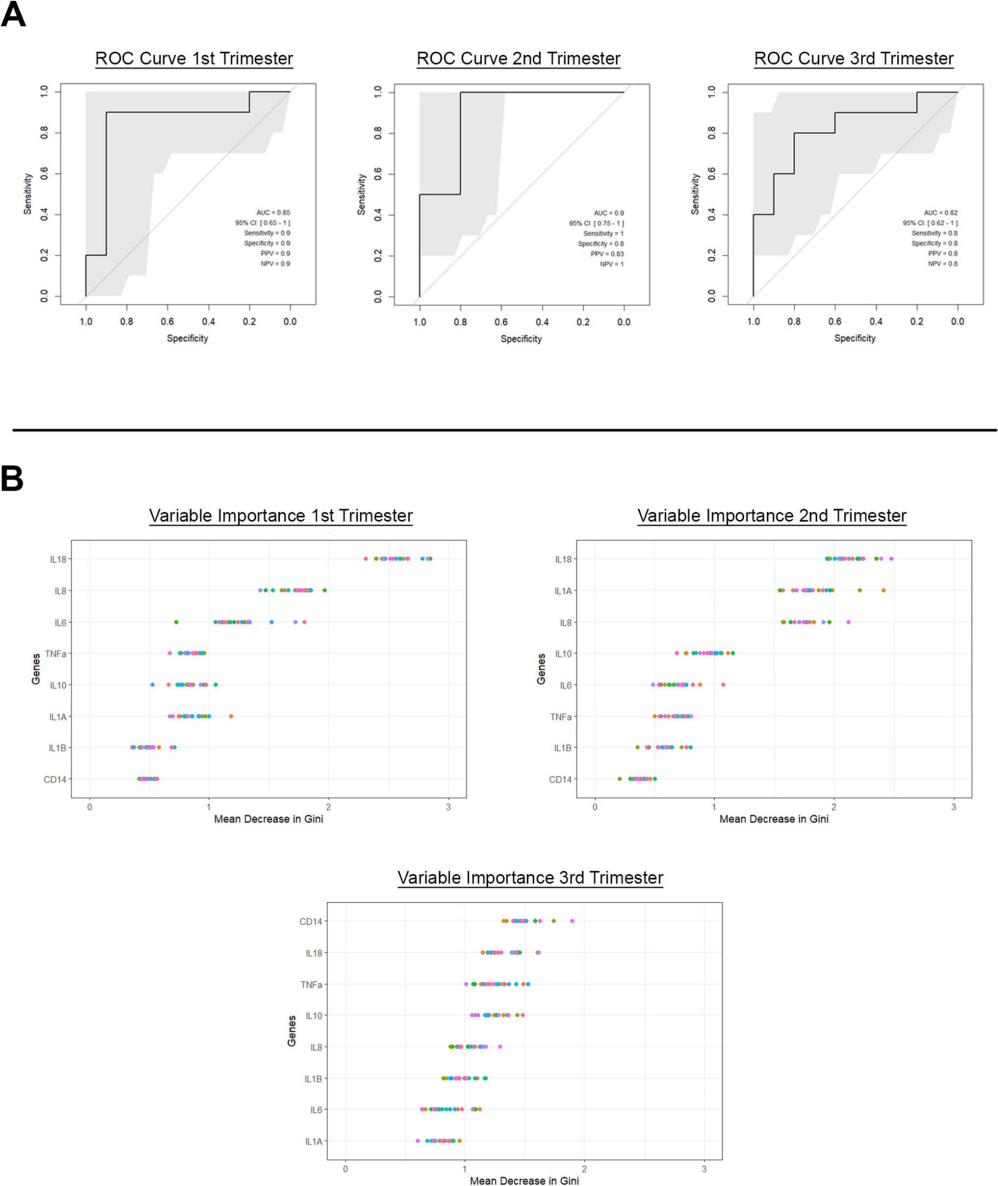
**A** AUC for inflammatory cfRNA signature in Human plasma samples 1 st**,** 2nd and 3rd Trimesters. **B** GINI plots for genes of variable importance across Human plasma samples for 1 st, 2nd and 3rd Trimesters

We performed targeted analyses utilising Random Forest Algorithm of the same eight cfRNA targets (*IL1α, IL1β, IL6, IL8, IL10, IL18, CD14, TNFα)* used in the chorioamnionitis study. Maternal blood samples were collected during the 1 st (at week 12) 2nd (at week 23) and 3rd (at week 32) trimesters of pregnancy. Serial (first, second, third trimester) plasma samples were collected from 10 patients that developed PE in the third trimester, and 10 patients that had uncomplicated term deliveries. Accordingly, each patient from the two groups had samples analysed at three different time points (1 st, 2nd and 3rd trimester). Maternal standardized Ct values for each individual cfRNA target are shown in the Supplementary Data. Random Forrest Classifier plots for maternal plasma gene target Cts are shown in Fig. 7A. The comparison of maternal plasma Ct feature sets for detecting inflammation associated with PE versus uncomplicated pregnancies showed a mean AUC of 0.85 (95% CI 0.65–1) in the 1 st trimester, 0.90 (95% CI 0.75–1) in the 2nd trimester, and 0.82 (95% CI 0.62–1) in the 3rd trimester (Fig. 7A). The sensitivity, specificity, PPV, NPV for maternal cfRNA prediction of PE at each trimester are displayed in Table 4. The relative contribution of each cfRNA gene target to the overall predictive power of detecting inflammation in the maternal plasma in the 1 st, 2nd and 3rd trimesters of patients with PE vs uncomplicated term pregnancy is shown in Fig. 7B. *IL18* was consistently one of the most important targets across all trimesters contributing the most variable importance for accurate prediction of inflammation in PE (Fig. 7B).

## Discussion

In this proof of principle study, we have drawn on data from the sheep model of pregnancy as well as human cohort samples and demonstrated that targeted analysis of cfRNA in maternal plasma has the potential to form the basis of a minimally invasive, highly accurate, predictive screening test for two inflammatory based pregnancy complications, namely chorioamnionitis and PE. Targeted cfRNA analysis of a sterile intrauterine inflammatory insult (LPS) at 2 d exposure (AUC 0.75) and 8 d exposure (AUC 1) had increased accuracy relative to clinical assessments used currently. These findings were further validated in the setting of a bacterial intrauterine inflammatory insult deriving from *Mycoplasma hominis* exposure at both an extremely early preterm gestation of 98 d gestation (AUC 0.84) and early preterm gestation of 128 d gestation (AUC 0.74). These findings are significant, as the current clinical criteria for antenatal diagnosis of clinical chorioamnionitis has poor predictive value and relies on a robust materno-fetal inflammatory response to be present, consistent with a sizable risk of fetal injury having already occurred.

Furthermore, in comparison to amniocentesis (the current gold standard for diagnosis of subclinical chorioamnionitis with a reported AUC of 0.82)[21], our approach has two main advantages. Firstly, targeted analysis of cfRNA in maternal plasma avoids the need for highly invasive amniocentesis which is often not clinically possible and is associated with a small risk of fetal demise. Secondly our approach provides a result within hours of the sample collection not days as is the case with amniotic fluid culture. With further development of this approach, it is expected that the time from maternal blood sample to result could be significantly shortened. Thereby allowing for earlier and more accurate diagnosis as well as screening of pregnant patients with suspected chorioamnionitis.

The extension of our findings developed in a sheep model of pregnancy and inflammation to the early prediction of PE in human samples further validates the utilisation of cfRNA in maternal plasma for prediction of multiple obstetric conditions related to inflammation. We demonstrated that targeted analysis of cfRNA accurately predicted PE across 1 st trimester (AUC 0.85), 2nd trimester (AUC 0.9) and 3rd trimester (AUC 0.81) in human maternal plasma samples. Our findings demonstrate increased accuracy compared to other approaches utilising cfRNA for the prediction of PE. For example Moufarrej et al*.* utilised a bulk sequencing approach to cfRNA analysis for the prediction of PE and demonstrated an AUC 0.71—0.74 in their validation cohorts between 5 and 16 weeks gestation[35]. Likewise, Rasmussen et al. utilising bulk sequencing of cfRNA, demonstrated a sensitivity of 75%, positive predictive value of 32.3% and AUC of 0.82 (95% CI ± 0.06) for the prediction of PE in the second trimester (16—27 weeks gestation)[36]. Our targeted cfRNA analysis of 8 genes has potential advantages compared to an untargeted bulk sequencing analysis in that this technique may be easier to translate into a bedside/laboratory test given the low cost, ease of analysis, and speed of data generation delivered using quantitative PCR as opposed to next generation sequencing. Thus, a maternal cfRNA approach may conceivably be used in a manner similar to that of bedside fetal fibronectin tests for the negative prediction of imminent preterm birth risk.

Of further interest is the relative importance of different gene targets across different gestations, which may provide further understanding to the pathophysiology of developing PE (Fig. 7B). Utilising our model, we demonstrate that *IL18* was highly important to the prediction of PE across all trimesters. *IL18* activates the NF-κB pathway which is involved in oxidative stress as well as inflammation and has been previously shown to be activated in placental tissue of PE patients[37]. Furthermore *IL-18* induces Fas-ligand mediated cytotoxicity which is thought to contribute to placental apoptosis in PE [38, 39]. Elevated plasma and placental levels of *IL18* have previously been demonstrated in patients with PE[40]. Of interest, *CD14* demonstrated the least importance in the 1 st and 2nd trimesters but in the 3rd trimester was the most important target for PE prediction. *CD14* is a pro-inflammatory cytokine that activates NF-κB and MAPK pathways and importantly has been linked to the development of arterial wall inflammation seen in atherosclerosis[41], a process which is also similarly seen in the spiral arteries of patients with PE.

Given that we have now validated the targeted analysis of cfRNA related to inflammation in human samples, it is hoped that these findings can also be translated into the early and accurate diagnosis of women with suspected chorioamnionitis.

### Strengths and Limitations

As with any proof-of-principle study, a number of limitations need to be taken into consideration in the interpretation of the data presented in this report. The sterile (endotoxin) and infectious (*Mycoplasma hominis)* chorioamnionitis data derive from the sheep model of pregnancy. Although this model system is widely accepted as a robust tool for the study of fetal physiology and development, differences in placentation and immunology between humans and the sheep naturally necessitate some caution in assessing the translatability of this work. At the same time, this sheep model of pregnancy allows for a highly standardised sterile and infectious inflammatory system to perform tightly controlled studies (magnitude, duration and stimulus of inflammatory exposure), removing much of the limiting experimental noise inherent to using human samples for proof-of-principle development studies.

Furthermore, we did not perform quantification (either by live culture or by qPCR) of total or viable *Mycoplasma hominis* load in amniotic fluid or in gestational tissues taken at delivery from the Infectious Chorioamnionitis Group animals. Accordingly, we are unable to correlate our data with the magnitude of infection at the time of delivery (a factor likely to impact the resultant inflammatory response) and are only able to conclude that changes in maternal cfRNA markers correlate with the introduction of *Mycoplasma hominis *into the amniotic fluid.

The inclusion of human samples for the PE analysis adds strength to this work as it demonstrates that the analysis system devised for this work is transferrable from the sheep to the human – both in terms of the primer targeting but also the sample preparation pipeline and the small volume of sample required to perform studies. The use of well-characterised PE samples against control samples also demonstrates that the technology developed herein has the ability to discriminate between a pro-inflammatory (i.e. pre-eclamptic) patient and a normal control patient in a clinical setting and well in advance of symptoms becoming clinically evident. It will be important now to validate these findings in a prospectively collected sample set, to ensure that the findings we report herein, which derive from a retrospective sample cohort, are reproducible without the benefit of an a priori understanding of disease status.

## Conclusion

Pathological inflammation in pregnancy is responsible for a material percentage of early births and is strongly associated with multi-system disease (notably the brain, lungs and GI tract) in preterm infants. At present, we lack accurate, rapidly deployable screening tools for the early detection of inflammation-associated pregnancy risk, hampering our ability to deploy effective interventions in a timely manner. The key findings of this report are that the analysis of maternal inflammation-associated cfRNA transcripts in the maternal placenta using a targeted qPCR approach may be used to identify intrauterine (chorioamnionitis) and systemic (pre-eclampsia) inflammatory processes strongly associated with adverse pregnancy outcomes for both mother and fetus. Additional studies to develop this technology will focus on samples drawn from large multi-centre and prospectively collected human cohorts.

## Data Availability

All data corresponding to figures and tables generated as well maternal standardized Ct values for each individual cfRNA target are available in supporting data deposited at figshare. Dataset. 10.6084/m9.figshare.27328659.v2. Further information and requests for resources and reagents should be directed to and will be fulfilled by the lead contact, Sean William David Carter (e0983855@u.nus.edu). This study did not generate new unique reagents. All physiological and haematological data presented in figures and text within this manuscript is available for review in the Supporting Data Values File. This paper does not report original code. Any additional information required to reanalyze the data reported in this paper is available from the lead contact upon request.

## Abbreviations

PE: Pre-eclampsia
cfRNA: Cell-free RNA
TNF: Tumour necrosis factor
sFLT1: Soluble fms-like tyrosine kinase-1
PlGF: Placental growth factor
LPS: Lipopolysaccharides
dGA: Days gestational age
IAI: Intraamniotic injection
Ct: Cycle threshold
AUC: Area under the receiver operating characteristic curve
CCU: Colour change units
PPV: Positive Predictive Value
NPV: Negative Predictive Value
WBC: White blood cell

## Declarations

### Inclusion and Diversity

We support inclusive, diverse, and equitable conduct of research.
